# Detección de infección por *Chlamydia trachomatis* en mujeres sexualmente activas en Venezuela

**DOI:** 10.7705/biomedica.6246

**Published:** 2022-09-02

**Authors:** Milagros Joya, Ricardo Heredia, Daniel Bastidas, Gilberto Bastidas

**Affiliations:** 1 Departamento de Microbiología, Facultad de Ciencias de la Salud, Universidad de Carabobo, San Diego, Carabobo, Venezuela Universidad de Carabobo Universidad de Carabobo San Diego Venezuela; 2 Escuela de Medicina, Facultad de Ciencias de la Salud, Universidad de Carabobo, San Diego, Carabobo, Venezuela Universidad de Carabobo Universidad de Carabobo San Diego Venezuela; 3 Departamento de Salud Pública y Centro de Investigaciones Médicas y Biotecnológicas, Facultad de Ciencias de la Salud, Universidad de Carabobo, San Diego, Carabobo, Venezuela Universidad de Carabobo Universidad de Carabobo Venezuela

**Keywords:** Clamydia trachomatis, infección, inmunoglobulina M**,** inmunoglobulina G**,** reacción en cadena de la polimerasa, epidemiología, Chlamydia trachomatis, infection, immunoglobulin M, immunoglobulin G, chain reaction polymerase, epidemiology

## Abstract

**Introducción.:**

La infección genital por *Chlamydia trachomatis* es una de las más frecuentes en el mundo. Cada año se registran cerca de 85 millones de nuevos casos de esta enfermedad, que cursa con graves complicaciones en la mujer y recién nacido.

**Objetivo.:**

Determinar las características clínico-epidemiológicas de la infección por *C. trachomatis* en mujeres venezolanas sexualmente activas.

**Materiales y métodos.:**

Es un estudio descriptivo, transversal y de campo, sustentado en la historia clínica y el examen físico, la detección de infección con la prueba inmunoenzimática con anticuerpos policlonales anti-LPS y la confirmación de los resultados con la de biología molecular. La muestra estuvo conformada por 100 mujeres sexualmente activas mayores de 12 años de edad, del estado Carabobo, Venezuela.

**Resultados.:**

La mayoría de las mujeres se encontraba entre los 20 y los 45 años de edad. En el 25 % de las mismas, se detectaron anticuerpos IgG anti-*C. trachomatis* y, en el 84 % de estas, se confirmó la infección mediante PCR; en ninguna de las mujeres se hallaron anticuerpos IgM anti-*C. trachomatis*.

**Conclusión.:**

La infección crónica predomina en las mujeres entre los 20 y los 45 años de edad; la prueba inmunoenzimática arrojó falsos positivos corroborados por PCR.

## Abstract (en)

**Introduction:**

Genital *Chlamydia trachomatis* infection is one of the most frequent in the world; about 85 million new cases of this pathology are registered each year, which causes severe complications in women and newborns.

**Objective:**

To determine the clinical-epidemiological characteristics of *C. trachomatis* infection in sexually active Venezuelan women.

**Materials and methods:**

Descriptive, cross-sectional, and field study based on the clinical history and physical examination, detection of infection with immunoenzymatic assay with anti-LPS polyclonal antibody and confirmation of results with molecular biology test. The sample consisted of 100 sexually active women over 12 years of age from Carabobo state, Venezuela.

**Results:**

The women were mostly between 20 and 45 years old, in 25% of them IgG antibodies to *C. trachomatis* were detected and in 84% of these the infection was confirmed by PCR, in none of the women IgM antibodies to *C. trachomatis* were found.

**Conclusion:**

Chronic infection characterizes women between 20 and 45 years of age; the immunoenzymatic test yielded false positives corroborated by PCR.

*Clamidia trachomatis* es una bacteria Gram negativa intracelular, de la cual se han identificado 18 serotipos hasta ahora. Las mujeres infectadas con esta bacteria pueden desarrollar síntomas muy leves o la infección genital por *C. trachomatis* puede persistir durante meses o años sin ser detectada, hasta la aparición, entre el 10 y el 40 % de los casos, de algunas de sus complicaciones por ascenso de la bacteria en el aparato genital. En este sentido, durante la gestación, puede producir embarazo ectópico, ruptura prematura de membranas y abortos espontáneos repetidos; en el recién nacido, ocasiona bajo peso al nacer, conjuntivitis, neumonía y aumento de la mortalidad perinatal; y, en la mujer, puede llevar a enfermedad inflamatoria pélvica e infertilidad ^1^^-^^4^.

La infección genital por *C. trachomatis* es una de las más frecuentes en el mundo. Cada año se registran cerca de 85 millones de nuevos casos, con una prevalencia global que oscila entre el 4,4 y el 6,6 % con infecciones recurrentes habituales ^1^^-^^4^. En Estados Unidos, se presentan anualmente entre 4 y 5 millones de casos nuevos de infección por *C. trachomatis*, de los cuales 2,6 millones ocurren en mujeres, 1,8 millones en varones y 250.000 en recién nacidos ^2^^,^^5^. Su costo estimado es de USD$ 2,4 billones ^6^ y se atribuye al tratamiento de las secuelas, muchas de ellas irreversibles; por tanto, se considera la infección de transmisión sexual más costosa después de la infección por HIV ^2^.

En Centroamérica, países como Costa Rica reportan una prevalencia de la infección hasta de 14,7 % en sujetos con factores de riesgo, pero sin relación entre la infección por *C. trachomatis* y el diagnóstico clínico sintomatológico, por lo que recomiendan implementar el diagnóstico por medio de pruebas de laboratorio (detección de antígenos o de ácidos nucleicos) ^7^^-^^9^. En Latinoamérica, mediante cultivos celulares de inóculos de muestras urogenitales, serología o ácido nucleico, la prevalencia de la infección varía entre 6 y 40 %, con mayor incidencia en adolescentes y en mujeres entre los 20 y los 25 años de edad ^1^^-^^4^.

Infortunadamente, Venezuela, y específicamente el estado Carabobo, cuenta con poca información epidemiológica sobre esta infección, por no considerarse una enfermedad de notificación obligatoria. Aunque se han hecho estudios, estos se han dirigido a describir las complicaciones obstétricas (ruptura prematura de membranas, amenaza de parto prematuro y trabajo de parto prematuro) y de los lactantes menores de 6 meses (sintomatología respiratoria o visual) derivadas de la infección del cuello uterino por *C. trachomatis*^10^^-^^12^, sin considerar el escrutinio poblacional en mujeres sexualmente activas.

El objetivo del presente estudio fue determinar la infección por *C. trachomatis* en mujeres sexualmente activas y describir algunas de sus características clínicas y epidemiológicas, con el fin de mostrar la magnitud real de la infección, porque el 90 % de las mujeres infectadas con este agente patógeno pueden permanecer asintomáticas y, por lo tanto, con los datos existentes se puede subestimar la verdadera prevalencia de la enfermedad y, en consecuencia, incrementar la probabilidad de complicaciones como el cáncer de cuello uterino, debido a su capacidad para inducir metaplasia al actuar como cofactor para el virus del papiloma humano (HPV) ^13^^-^^15^.

## Materiales y métodos

### Población y muestra

Para el abordaje de la presente investigación, se recurrió al estudio descriptivo, transversal y de campo. La muestra incluyó 100 mujeres, tamaño obtenido a partir de la frecuencia esperada de mujeres con infección por *C. trachomatis* (tomada como referencia de los estudios en Suramérica) ^16^ y del tamaño de la población femenina mayor de 12 años de edad y sexualmente activa (con relaciones sexuales, al menos, una vez por semana). Para el muestreo probabilístico se calculó el intervalo numérico para la selección de la muestra y se incluyeron todas aquellas mujeres sexualmente activas que, de forma voluntaria, aceptaron la realización de la historia médica, el examen físico y la toma de muestra sanguínea y de exudado endocervical, y se excluyeron a las que tenían antecedentes de terapia antibiótica sistémica o local en los 30 días previos a la evaluación.

### Historia clínica y examen físico

Antes de la elaborar la historia clínica, se obtuvo el consentimiento informado de cada mujer previa explicación de los objetivos de la investigación, así como también de los padres o representantes legales de las menores que participaron. También, se consiguió el aval del comité de bioética de los centros hospitalarios donde fueron reclutadas las mujeres (en el Ambulatorio Urbano Tipo II Dr. Miguel Franco del municipio Naguanagua y en el Hospital González Plaza del municipio Valencia).

Se garantizó a cada participante la confidencialidad de la información obtenida por medio de la historia clínica, el examen físico y las pruebas de laboratorio. En la historia clínica se incluyeron datos sociodemográficos como edad, estado civil, estrato socioeconómico y nivel educativo, además de los antecedentes ginecoobstétricos.

Según la edad, se conformaron tres grupos etarios: adolescentes, entre los 12 y los 19 años de edad; mujeres en edad reproductiva, entre los 20 y los 45 años; y, mujeres menopáusicas y mayores, entre los 46 y los 60 años. Para la clasificación según el estrato socioeconómico, se tuvieron en cuenta: profesión y nivel de instrucción del jefe de familia, fuente de ingreso familiar, comodidad de la vivienda y aspectos de la zona geográfica donde se ubica la vivienda; los estratos fueron cuatro: I, alta calidad de vida; II, moderada calidad de vida; III, baja calidad de vida, y IV, pobreza extrema.

### Toma de la muestra de sangre

De cada mujer se obtuvieron entre 2 y 3 ml de sangre, mediante venopunción con jeringa estéril. Las muestras sanguíneas se colocaron en un tubo de ensayo sin anticoagulante para separar el suero. Finalmente, el suero de cada paciente se almacenó a -80 °C hasta practicar las pruebas.

### Toma de muestra cérvico-vaginal

Debido a que la bacteria infecta específicamente a las células columnares y la infección es intracelular, las muestran se obtuvieron raspando el sitio apropiado, el epitelio columnar del endocérvix, previa visualización del cuello uterino con la ayuda de un espéculo, sin usar lubricantes. El hisopo se introdujo en la cavidad vaginal hasta el interior del cuello uterino la distancia suficiente para que no se vea la punta de algodón, y se hizo el raspado riguroso del epitelio cervical, rotando por algunos segundos el hisopo. El hisopo con el exudado endocervical se colocó en 2 ml de medio de transporte de sacarosa-fosfato (2SP) en hielo, para ser congeladas a -80 °C.

### Detección de infección por Chlamydia trachomatis

*Estudio serológico*

Se emplearon pruebas inmunoenzimáticas directas con anticuerpo policlonal anti-LPS, por ser la molécula más abundante en la pared de *C. trachomatis*, donde un anticuerpo conjugado a una enzima cambia el color de un sustrato, de incoloro a coloreado, fenómeno que fue medido en un espectrofotómetro. Estas pruebas incluyeron un reactivo bloqueador, y un anticuerpo monoclonal anti-LPS para el epítopo al cual se une el anticuerpo policlonal, con el fin de confirmar los resultados positivos. En este estudio, se empleó un estuche comercial (BIOLINE Diagnostic Inc., USA) ^17^. Las pruebas incluyeron controles negativos y positivos. La intensidad del color generado es proporcional a la cantidad de anticuerpo de IgM específica contra *C. trachomatis*, y se midió espectrofotométricamente a 450 nm, con un espectrofotómetro Titertek Multiskan™ (Phoenix Equipment Inc., USA).

### Estudio de biología molecular

Para la extracción del ADN, la muestra se suspendió en 1 ml de PBS. Enseguida, se añadieron 100 μΙ de solución tampón proteolítica (320 mM de sucrosa, 10 Mm Tris (pH=8), 5 mM de MgCl_2_, 1 % SDS y 40 mg/ml de proteinasa K) a 100 μΙ de la solución con el ADN. Después de la incubación a 55 °C durante 2 horas, la solución se mezcló con 200 μl de fenol equilibrado y se centrifugó a 8.000*g* durante cinco minutos. La fase acuosa se mezcló con 200 μl de cloroformo y se centrifugó de nuevo a 8.000*g* durante cinco minutos. El ADN se precipitó con 400 μl de etanol absoluto y acetato de sodio 2 M a -20 °C durante 1 hora. Después de secar los tubos, el ADN se precipitó de nuevo con 100 μl de etanol al 70 % a -20 °C durante una hora y, después, se disolvió en 50 μl de solución tampón TE. El ADN extraído se almacenó a -20 °C hasta la práctica de la prueba molecular.

Para la reacción en cadena de la polimerasa (PCR), se emplearon las secuencias de los cebadores de MWG-Biothech™, Alemania (KL 5 5' **-** TCC GGA GCG AGT TAC GAGA; KL2 5’AAT CAT TGC CGG GGA TTG GT), cuyo blanco de amplificación es el plásmido ORF 2 de 7,5 kb y el tamaño de la amplificación es de 241 bp. La reacción de amplificación múltiple se realizó en un volumen de 25 μl que contenía: 0,2 mM de dNTP, 2,5 mM de 1,5 mM MgCl_2_, 50 pmol de cada cebador, solución tampón 1X de PCR, 3 μl de ADN. y 1,6 U de Taq polimerasa. La reacción se realizó durante 35 ciclos bajo las siguientes condiciones: 45 s a 95 °C, 30 s a 50 °C y 45 s a 72 °C. Después del último ciclo, se hizo una etapa final de 10 minutos a 72 °C para completar la elongación. Los productos de amplificación se visualizaron y fotografiaron bajo luz ultravioleta después de la electroforesis durante 45 minutos a 100 V en un gel de agarosa al 1 % con bromuro de etidio ^18^^,^^19^.

### Análisis estadístico

La información obtenida se procesó en una base de datos y se analizó con el programa SPSS™, versión 20 (IBM, Armonk, New York) para las evaluaciones de tipo estadístico. De las variables categóricas, se obtuvieron las frecuencias y proporciones, se estimó la prevalencia y se calcularon los intervalos de confianza de 95 % binomiales correspondientes. Se realizó inferencia estadística entre variables de interés, con la distribución de ji al cuadrado en la prueba de hipótesis.

## Resultados

De las 100 muestras examinadas de mujeres sexualmente activas, el 63 % se ubicaba en el grupo de edad comprendido entre los 20 y los 45 años (etapa reproductiva), el 29 % de ellas se encontraba ya en la posmenopausia, tenían entre 46 y 60 años edad, y el 8 % estaba en la fase de adolescencia, aún con inmadurez de su aparato genital (p<0,0001, con diferencia significativa entre las que tienen entre 20 y 45 años y la suma de los otros dos grupos de edad).

La edad promedio del grupo de mujeres de la muestra fue de 37,3 ± 11,3 años; la de menor edad tenía 14 años y la de mayor edad, 58. Entre las 100 mujeres, fueron más las solteras (68 %) que las casadas (27 %), únicas que refieren pareja sexual estable, y que las divorciadas (5 %) (p<0,0000001, con diferencia significativa entre mujeres solteras y la suma de las casadas y divorciadas).

Los estratos socioeconómicos predominantes en la muestra de mujeres fueron el II (moderada calidad de vida) (52/100) y el III (baja calidad de vida) (39/100), sin diferencia significativa entre ambos estratos (p=0,05). Respecto al nivel educativo, la mayoría tenía educación secundaria incompleta (39/100) con diferencia significativa entra esta y los niveles de primaria (p<0,01), universitaria (p<0,0001) y secundaria completa (p<0,00001) (cuadro 1).

**Cuadro 1:** Características sociodemográficas de la muestra de mujeres sexualmente activas del estado Carabobo, Venezuela, en quienes se determinó infección por *Chlamydia trachomatis.*

		n	Media	Desviación estándar
Edad (años)*				
	12 a 19	8	16,8	2,5
	20 a 45	63	34,1	7,5
	46 a 60	29	50,1	3,4
Estado civil**			n	
	Soltera		68	
	Casada		27	
	Divorciada		5	
Estrato socioeconómico			n	
	I		2	
	II		52	
	III		39	
	IV		7	
Nivel educativo***			n	
	Educación primaria		26	
	Educación secundaria incompleta		39	
	Educación secundaria completa		16	
	Universitaria		19	

Ji al cuadrado: * p<0,0001, con diferencias significativas entre mujeres de 20 a 45 años de edad, y la suma de las que están entre 12 y 19 o entre 46 y 60 años

** p<0,00000001, con diferencias significativas entre las mujeres solteras y la suma de las casadas más las divorciadas

*** p<0,01, con diferencia significativa entre educación secundaria incompleta y primaria; p<0,0001, con diferencia significativa entre educación secundaria incompleta y universitaria; p<0,00001, con diferencia significativa entre educación secundaria incompleta y completa.

De las 100 mujeres de la muestra, 57 empleaban algún método anticonceptivo, sin diferencia significativa entre las que utilizaban algún tipo de anticoncepción y las que no lo hacían (p=0,047). Los métodos de barrera, únicos que protegen contra la infección por *C. trachomatis*, ocupan el penúltimo (3 %) lugar entre las preferencias, con diferencia significativa entre este y el resto de métodos anticonceptivos (p<0,0000).

El 36 % de las mujeres presentaron infecciones de transmisión sexual, exclusivamente por virus del papiloma humano y candidiasis. Del total de mujeres que lograron embarazos (87/100), 37,9 % lo hicieron cuatro o más veces. De las mujeres que lograron algún embarazo, en el 22,9 % (20/87) se produjo aborto; el embarazo ectópico y las lesiones premalignas del cuello uterino, se presentaron cada una en el 2 % del total de la muestra (cuadro 2).

**Cuadro. 2:** Antecedentes ginecoobstétricos de la muestra de mujeres sexualmente activas del estado Carabobo, Venezuela, en quienes se determinó infección por *Chlamydia trachomatis*

Antecedentes		n	%
Anticoncepción			
	Quirúrgicos	28,0	28,0
	Oral	15,0	15,0
	Dispositivo intrauterino	10,0	10,0
	Barreras*	3,0	3,0
	Inyectables	1,0	1,0
	Ninguno	43,0	43,0
Infección de transmisión sexual			
	Sí	36	36,0
	No	64	64,0
Embarazos			
	0	13	13,0
	1	10	10,0
	2	18	18,0
	3	26	26,0
	≥4	33	33,0
Abortos			
	1	12	12,0
	2	7	7,0
	3	1	1,0
Embarazos ectópicos		2	2,0
Lesiones premalignas de cuello uterino		2	2,0

Ji al cuadrado: *p< 0,0000, con diferencias significativas entre el empleo de métodos de barrera y el del resto de herramientas anticonceptivas

En la muestra estudiada, no hubo casos positivos para anticuerpos IgM anti-*C. trachomatis*. La proporción de casos positivos para anticuerpos IgG anti-*C. trachomatis* fue del 25 % (25/100 mujeres), principalmente en aquellas con edades comprendidas entre los 20 y los 45 años (75 %; 9/12), con diferencia significativa entre este grupo de edad y la unión de los otros dos (p<0,01) (cuadro 3). De las 25 muestras de suero que resultaron con anticuerpos IgG anti-*C. trachomatis*, el 84 % (21/25) también resultaron positivas para este agente infeccioso con la reacción de amplificación de los ácidos nucleicos, sin diferencia significativa entre las pruebas diagnósticas (p=0,53) (cuadro 4).

**Cuadro. 3:** Casos positivos o negativos para anticuerpos IgG anti-*Chlamydia trachomatis* en mujeres sexualmente activas del estado Carabobo, Venezuela

IgG	Grupos de edad						Total	
	12 a 19		20 a 45		46 a 60			
	n	%	n	%	n	%	n	%
Positiva *	1	1,0	17	17,0	7	7,0	25	25,0
Negativa	7	7,0	46	46,0	22	22,0	75	75,0
Total	8	8,0	63	63,0	29	29,0	100	100,0

Ji al cuadrado: * p< 0,01, con diferencias significativas entre mujeres de 20 a 45 años de edad, y la suma de las que están entre 12 y 19 o entre 46 y 60 años

**Cuadro 4:** Determinación por PCR de infección por *Chlamydia trachomatis* en mujeres sexualmente activas del estado Carabobo, Venezuela

Grupo de edad	Resultado PCR					
	Positivo		Negativo		Total	
	n	%	n	%	n	%
12 a 19	1	4,0	0	0,0	1	4,0
20 a 45	15	60,0	2	8,0	17	68,0
46 a 60	5	20,0	2	8,0	7	28,0
Total	21	84,0	4	16,0	25	100,0

## Discusión

La prevalencia encontrada de infección por *C. trachomatis* en mujeres sexualmente activas, se ubica dentro del rango esperado para Latinoamérica ^20^^,^^21^. No obstante, en el interior del país, la prevalencia de la infección por *C. trachomatis* puede considerarse alta en relación con la hallada en otros estados como Zulia (10,4 %), mediante el diagnóstico molecular en 105 pacientes que acudieron a un servicio de salud ambulatorio rural de la entidad política territorial ^22^^,^^23^.

Las diferencias de prevalencia obtenidas en los grupos de edad de estudio son también congruentes con otras investigaciones. En el presente estudio, el grupo de edad más afectado fue el de 20 a 45 años (68%; 17/25), en comparación con el grupo de 12 a 19 años y el de 46 a 60 años. También, se evidencia que apenas el 4,8 % (1/21) de las mujeres con 19 años o menos tienen infección por *C. trachomatis*, a pesar de que en ellas se describe un comportamiento sexual de gran riesgo (menor empleo de preservativos); en el histológico, es evidente la ectopia cervical (exposición de las células escamo-columnares a *C. trachomatis*) y mayor tendencia a cambiar de pareja (se reconoce que la probabilidad de que un individuo se infecte se incrementa con el cambio frecuente de pareja) ^8^^,^^24^.

La influencia sobre la infección por *C. trachomatis* de la precariedad económica y afectiva, del bajo nivel educativo y de la juventud reportada en otros estudios, debe ser nuevamente abordada en intervenciones futuras de la población venezolana, toda vez que no fue posible determinarlas en este estudio. En la muestra, las mujeres entre 46 y 60 años tienen con gran frecuencia infección por *C. trachomatis* (7%; 7/100); la mayoría eran solteras o divorciadas, sin un compromiso que las mantenga unidas a una sola pareja sexual y, por lo tanto, no debe descartarse la existencia de distintos contactos sexuales ^12^.

En ningún caso se detectaron anticuerpos IgM anti-*C. trachomatis*, quizás porque este anticuerpo es un marcador inexacto de infección aguda o porque las mujeres estudiadas pudieron haber tenido infecciones previas con *C. trachomatis* o con otra bacteria del mismo género como *C. pneumoniae*, con una reacción inmunitaria amnésica a una exposición reciente. Como se dijo anteriormente, con el diagnostico serológico se identificaron 25 muestras con anticuerpos IgG anti-*C. trachomatis*; sin embargo, con la PCR solo fue posible corroborar la infección en 21 de las mismas. La mayor proporción de muestras positivas por técnica diagnóstica inmunoenzimática pudo deberse a reactividad cruzada con *C. pneumoniae* o *C. psittaci*, porque los epítopos específicos para el género están presentes en todos los antígenos que se derivan de estas bacterias, a lo que se suma la persistencia de anticuerpos (IgG) por largos periodos y su lenta declinación ^18^^,^^25^^,^^26^.

Este estudio tiene limitaciones relacionadas con el tamaño de la muestra, que impide el estudio de los factores determinantes de la infección e, incluso, de las complicaciones que se producen tras ella. Asimismo, aunque la muestra es representativa de los centros de salud participantes, los resultados obtenidos no pueden extrapolarse a todo el estado Carabobo y, menos aun, a Venezuela, a pesar de que este sesgo puede resultar minimizado por la cobertura universal de la población que atienden los centros asistenciales. Por lo tanto, debe considerarse como un reporte preliminar y la base para investigaciones de mayor complejidad y duración.

## Conclusiones

La prevalencia encontrada de infección por *C. trachomatis* en mujeres sexualmente activas, se ubica dentro del rango esperado para Latinoamérica. En el presente estudio, las mujeres más afectadas tenían entre 20 y 45 años de edad; en ellas se detectó exclusivamente infección crónica por *C. trachomatis.* Asimismo, las pruebas inmunoenzimáticas arrojaron falsos positivos corroborados por prueba de biológica molecular.

Este trabajo se constituye en un reporte preliminar para estudios con mayor tamaño poblacional e inclusión de más variables, con el objetivo de mostrar la magnitud y trascendencia real de las infecciones por *C. trachomatis* en las poblaciones venezolanas.

## References

[1] Davey J, Shull H, Billings J, Wang D, Adachi K, Klausner J. (2016). Prevalence of curable sexually transmitted infections in pregnant women in low- and middle-income countries from 2010 to 2015: A systematic review. Sex Transm Dis.

[2] Witkin S, Minis E, Athanasiou A, Leizer J, Linhares I. (2017). Chlamydia trachomatis: The persistent pathogen. Clin Vaccine Immunol.

[3] Ahmed N, Sharma A, Satpathy G, Titiyal J, Tandon R, Agarwal T (2018). Chlamydia trachomatis antigen positivity in patients with different ocular manifestations over 8 years. J Glob Infect Dis.

[4] Janssen K, Dirks J, Dukers-Muijrers N, Hoebe C, Wolffs P. (2018). Review of Chlamydia trachomatis viability methods: Assessing the clinical diagnostic impact of NAAT positive results. Expert Rev Mol Diagn.

[5] Bianchi S, Frati E, Canuti M, Colzani D, Fasoli E, Amendola A (2016). Molecular epidemiology and genotyping of Chlamydia trachomatis infection in a cohort of young asymptomatic sexually active women (18-25 years) in Milan, Italy. J Prev Med Hyg.

[6] Ditkowsky J, Shah K, Hammerschlag M, Kohlhoff S, Smith-Norowitz T. (2017). Cost-beneft analysis of Chlamydia trachomatis screening in pregnant women in a high burden setting in the United States. BMC Infect Dis.

[7] García A, Araúz P, Taylor L, Moraga M, Herrera G. (2005). Infección por Chlamydia trachomatis en un grupo de mujeres de alto riesgo. Trabajadoras del sexo en Costa Rica. Rev Costarric Cienc Méd.

[8] Joya M, Joya A, Sequera M, Arteaga E, Bastidas G. (2014). Prevalencia de la infección por Chlamydia trachomatis en hombres infértiles. MedULA.

[9] Goggins E, Chamberlain A, Kim T, Young M, Jamieson J, Haddad L. (2020). Patterns of screening, infection, and treatment of Chlamydia trachomatis and Neisseria gonorrhea in pregnancy. Obstet Gynecol.

[10] Arteaga B, Messina C, Rocha E, Chacón E, Rodulfo F. (1996). Factores de riesgo de la infección por Chlamydia trachomatis en lactantes menores de 6 meses: estudio prospectivo Hospital Dr. Leopoldo Manrique Terrero, Caracas, Venezuela. Marzo 1995-octubre 1996. Arch Venez Pueric Pediatr.

[11] Joolayi F, Navidifar T, Mohammad R, Amin M. (2017). Comparison of Chlamydia trachomatis infection among infertile and fertile women in Ahvaz, Iran: A case-control study. Int J Reprod Biomed (Yazd).

[12] Kiguen A, Marramá M, Ruiz S, Estofan P, Venezuela R, Mosmann J (2019). Prevalence, risk factors and molecular characterization of Chlamydia trachomatis in pregnant women from Córdoba, Argentina: A prospective study. PLoS ONE.

[13] Zambrano A, Muñoz B, Mkocha H, Dize L, Gaydos C, Quinn T (2017). Measuring trachomatous inﬂammation-intense (TI) when prevalence is low provides data on infection with Chlamydia trachomatis. Invest Ophthalmol Vis Sci.

[14] Ministerio del Poder Popular para la Salud (2018). Alertas epidemiológicas. Dirección de Epidemiología y Análisis Estadístico. Dirección de Vigilancia Epidemiológica.

[15] Olson-Chen C, Balaram K, Hackney D. (2018). Chlamydia trachomatis and adverse pregnancy outcomes: Meta-analysis of patients with and without infection. Matern Child Health J.

[16] de Cetina T, Polanco-Reyes L, Fernández-González V, Ruiz-García S. (2003). Infección por Chlamydia trachomatis en usuarias de dos clínicas de planifcación familiar. Salud Pública Méx.

[17] Poussin M, Fuentes V, Corbel C, Prin L, Eb F, Orfla J. (1997). Captura-ELISA; un nuevo ensayo para la detección de anticuerpos de inmunoglobulina isotipo M utilizando antígeno Chlamydia trachomatis. J Immunol Methods.

[18] Rawre J, Juyal D, Dhawan B. (2017). Molecular typing of Chlamydia trachomatis: An overview. Indian J Med Microbiol.

[19] Frej-Mądrzak M, Gryboś A, Gryboś M, Teryks-Wołyniec D, Jama-Kmiecik A, Sarowska J (2018). PCR diagnostics of Chlamydia trachomatis in asymptomatic infection by women. Ginekol Pol.

[20] Huai P, Li F, Chu T, Liu D, Liu J, Zhang F. (2020). Prevalence of genital Chlamydia trachomatis infection in the general population: A meta-analysis. BMC Infect Dis..

[21] Giuliano A, Denman C, Guernsey J, Navarro J, Ortega L, Djambazov B (2001). Design and results of the USA-Mexico border human papillomavirus (HPV), cervical dysplasia, and Chlamydia trachomatis study. Rev Panam Salud Pública.

[22] Arráiz R, Ginestre P, Perozo M, Castellano G, Urdaneta B, García G. (2007). Molecular diagnosis and Chlamydia trachomatis infections prevalence in symptomatic and asymptomatic patients of a population of the Zulia State, Venezuela. Rev Chilena Infectol.

[23] Arraiz N, Ginestre M, Castellano M, Perozo A, Urdaneta B. (2006). Detección de Chlamydia trachomatis en muestras de hisopado endocervical por inmunoﬂuorescencia directa y reacción en cadena de la polimerasa. Rev Soc Ven Microbiol.

[24] Czerwinski M, Niedzwiedzka-Stadnik M, Zielicka-Hardy A, Tomusiak A, Sadkowska-Todys M, Zielinski A (2018). Genital Chlamydia trachomatis infections in young adults a school-based bio-behavioural study in urban areas, Poland, 2012 to 2015. Euro Surveill.

[25] Banniettis N, Thumu S, Weedon J, Chotikanatis K, Szigeti A, Hammerschlag M (2017). Seroprevalence of Chlamydia trachomatis in inner-city children and adolescents-implications for vaccine development. Sex Transm Dis.

[26] Frej-Mądrzak M, Gryboś A, Gryboś M, Teryks-Wołyniec D, Jama-Kmiecik A, Sarowska J (2018). PCR diagnostics of Chlamydia trachomatis in asymptomatic infection by women. Ginekol Pol.

